# A new molecular risk pathway for postpartum mood disorders: clues from steroid sulfatase–deficient individuals

**DOI:** 10.1007/s00737-020-01093-1

**Published:** 2020-11-20

**Authors:** Harish Thippeswamy, William Davies

**Affiliations:** 1 Department of Psychiatry, National Institute of Mental Health and Neuro Sciences (NIMHANS), Bangalore, India; 2 Centre for Neuropsychiatric Genetics and Genomics and Division of Psychological Medicine and Clinical Neurosciences, School of Medicine, Cardiff University, Cardiff, UK; 3 School of Psychology, Cardiff University, Tower Building, 70, Park Place, Cardiff, CF10 3AT UK; 4 Neuroscience and Mental Health Research Institute, Cardiff University, Cardiff, UK

**Keywords:** Calcium signalling, Cellular Communication Network Factor, Oestrogen, Phosphodiesterase 10A, Psychosis, Transient Receptor Potential Cation Channel Subfamily M Member 3 (TRPM3)

## Abstract

Postpartum mood disorders develop shortly after childbirth in a significant proportion of women. These conditions are associated with a range of symptoms including abnormally high or low mood, irritability, cognitive disorganisation, disrupted sleep, hallucinations/delusions, and occasionally suicidal or infanticidal ideation; if not treated promptly, they can substantially impact upon the mother’s health, mother-infant bonding, and family dynamics. The biological precipitants of such disorders remain unclear, although large changes in maternal immune and hormonal physiology following childbirth are likely to play a role. Pharmacological therapies for postpartum mood disorders can be effective, but may be associated with side effects, concerns relating to breastfeeding, and teratogenicity risks when used prophylactically. Furthermore, most of the drugs that are used to treat postpartum mood disorders are the same ones that are used to treat mood episodes during non-postpartum periods. A better understanding of the biological factors predisposing to postpartum mood disorders would allow for rational drug development, and the identification of predictive biomarkers to ensure that ‘at risk’ mothers receive earlier and more effective clinical management. We describe new findings relating to the role of the enzyme steroid sulfatase in maternal postpartum behavioural processes, and discuss how these point to a novel molecular risk pathway underlying postpartum mood disorders. Specifically, we suggest that aberrant steroid hormone–dependent regulation of neuronal calcium influx via extracellular matrix proteins and membrane receptors involved in responding to the cell’s microenvironment might be important. Testing of this hypothesis might identify novel therapeutic targets and predictive biomarkers.

## A brief introduction to postpartum mood disorders

Following childbirth, women can present with a number of mood conditions, ranging in prevalence and severity. The ‘baby blues’, which affects ~ 40% of new mothers, is associated with comparatively mild depressive symptoms which typically resolve spontaneously within a relatively short timeframe (Rezaie-Keikhaie et al. 2020). The symptoms of postpartum depression (PPD) are similar to, but more severe and more prolonged than, those associated with the baby blues; they can onset up to one year post-childbirth and often require medical treatment (Meltzer-Brody et al. 2018b). The point prevalence of postpartum depression has been estimated as being between 13 and 20% within the general population, and up to 35% of individuals with a previous psychiatric history; notable psychosocial risk factors include young age and lack of social support, a personal or family history of depression, and exposure to adverse early-life adverse events (Meltzer-Brody et al. 2018b). Perinatal anxiety, often associated with abnormal concerns about the welfare of the newborn baby, is thought to affect ~ 20% of all mothers (Fawcett et al. 2019). Furthermore, childbirth may precipitate a recurrence of bipolar illness, especially amongst women with bipolar disorder and a past history of perinatal depression or affective psychosis (Di Florio et al. 2018). Postpartum psychosis (PP) is the rarest and arguably most severe of the postpartum mood disorders, and is thought to affect 0.89–2.6 of every 1000 mothers (VanderKruik et al. 2017). PP is associated with significant mood symptoms (including depression and mania), with cognitive disorganisation and confusion, with hallucinations and delusions (often baby-orientated), and with substantial changes to sleep patterns (Bergink et al. 2016); a prior diagnosis of bipolar disorder is a strong risk factor for PP and women who experience mania following sleep loss may be at particular risk (Lewis et al. 2018). However, nearly two-thirds of women who experience PP have no prior psychiatric history (Blackmore et al. 2013). Postpartum mood disorders are frequently comorbid with other medical conditions including thyroid disorders (Bergink et al. 2011; Guintivano et al. 2018; Minaldi et al. 2020) and pre-eclampsia (Bergink et al. 2015; Caropreso et al. 2019).

Despite preventative strategies being employed for women at known risk of postpartum mood disorders, some new mothers will nevertheless be affected. Treatments for such disorders are often reasonably effective if administered shortly after symptom onset. These include pharmacotherapy (antidepressant, mood-stabilising, anxiolytic, sedative, and antipsychotic drugs administered alone or in some combination), electroconvulsive therapy, psychological therapies, and family/social support; in rare cases where the disorder has an underlying autoimmune basis, interventions targeting the immune system may be efficacious (Meltzer-Brody et al. 2018b; Reddy et al. 2018). However, these approaches are not universally effective and can result in unpleasant side effects, and caution is warranted when taking some medications prophylactically or whilst breastfeeding given the risk of teratogenicity to the foetus in utero, or of consumption by the feeding infant, respectively (Lusskin et al. 2018; Poels et al. 2018).

A significant biological contribution to postpartum mood disorder risk is likely: these conditions are closely temporally linked to the maternal physiological perturbations following childbirth, they are associated with a similar range of symptoms and prevalence across differing cultures and societies, and they tend to run in families with individuals affected by one episode at risk for subsequent births. Much work investigating the biological basis of postpartum mood disorders has focussed upon physiological systems which are in flux during pregnancy (particularly late pregnancy) and into the puerperium. After the steroidogenic placenta is expelled and as gonadal production of steroid hormones stabilises following parturition, levels of oestrogens, progesterone, and the progesterone metabolite allopregnanolone plummet acutely; these hormones typically act on the limbic regions of the brain involved in emotional and cognitive processing, motivation, and arousal, modulating neurotransmitter (notably gamma-aminobutyric acid receptor type A (GABA_A_) receptor) function (Meltzer-Brody et al. 2018b). Abnormal sensitivity to this steroid hormone depletion, perhaps mediated in part via GABAergic maladaptation, has been proposed to account for postpartum mood disorder risk (Meltzer-Brody et al. 2018b). Recent studies linking allopregnanolone levels to depressive-anxiety symptoms, and showing beneficial effects of allopregnanolone administration in women with postpartum depression, emphasise the potential importance of this particular compound (Deligiannidis et al. 2016; Meltzer-Brody et al. 2018a; Paul et al. 2020). Links between postpartum mental health risk and levels/activity of other reproductive, stress, and thyroid hormones have also been proposed, but these appear less consistent and are of more uncertain impact (Meltzer-Brody et al. 2018b). Immune perturbation in the postpartum period, specifically an imbalance between pro- and anti-inflammatory factors, has also been suggested as a candidate risk mechanism (Bergink et al. 2013; Kumar et al. 2017; Meltzer-Brody et al. 2018b; Dazzan et al. 2018); this imbalance may be related to changes in steroid hormone levels (Bouman et al. 2005) or may be aetiologically distinct. In a diathesis model, the underlying sensitivity to acute postpartum hormonal and immune changes may be partially explained by (epi)genetic factors. To date, (epi)genetic studies in these conditions have focussed upon candidate genes, or have been underpowered to robustly detect risk variants with meaningful effects; however, animal model and follow-up clinical work has indicated promising hormone-sensitive epigenetic signatures around the *HP1BP3* and *TTC9B* loci that may be associated with postpartum depression (Payne et al. 2020). With the establishment of large consortia and better-powered genome-wide screens, over the next few years, such variants may be identified.

Animal models in which experimental variables can be systematically altered, and in which access to relevant brain tissue is not a concern, may be informative for understanding the biology of postpartum mood conditions; such models include selective breeding models, behavioural models where pregnant mothers are repeatedly exposed to stress throughout pregnancy and the postpartum period, or pharmacological models involving the administration/withdrawal of stress and steroid hormones (Perani and Slattery 2014); however, mammalian models such as rodents differ significantly from humans in terms of their reproductive function and the extent to which findings can be generalised across species is unclear (Martin 2007).

Clarifying the biological factors underlying postpartum mood disorder risk through parallel clinical and animal model studies will increase the likelihood of identifying targets for more effective medications with fewer and/or less severe side effects, of understanding variability in individual treatment response to existing drugs, and of characterising biomarkers with possible predictive potential. Below, we argue that the enzyme steroid sulfatase may be a mediator of maternal behaviour, and that the downstream pathways it influences may be disrupted in idiopathic postpartum mood disorders.

## Steroid sulfatase: Its function and expression pattern

Steroid sulfatase (STS) is an enzyme encoded by the X-linked *STS* gene located at Xp22.31 (Reed et al. 2005). It appears to be the sole mediator of steroid desulfation in mammals, cleaving sulfate groups from a variety of relatively inert, water-soluble compounds to convert them to more active, less soluble moieties which can act as precursors for a variety of oestrogens and androgens (Mueller et al. 2015).

In the developing human foetus, *STS* is expressed in numerous regions of the brain (most highly in the hypothalamus and the anterior lobe of the pituitary gland, thalamus, cortex, cerebellar neuroepithelium, and basal ganglia), as well as in the thyroid gland (Stergiakouli et al. 2011) and the placenta (Geyer et al. 2017); in adult tissues, STS continues to be expressed in the brain (most highly in the frontal cortex) and in the thyroid gland (GTEx Portal 2020). There is some evidence that in mammals, brain and peripheral STS protein levels/activity increase towards late pregnancy and into the postpartum period (Davies 2018). In the brain, sulfated and free steroids have partially dissociable activities at membrane proteins including GABA_A_ and *N*-methyl-d-aspartate (NMDA) receptors which act as ligand-gated chloride and sodium/calcium ion channels respectively (Davies 2012); levels of these compounds also correlate with markers of immune function during pregnancy and into the postpartum period (Tagawa et al. 2004a; Tagawa et al. 2004b).

As STS is expressed in relevant tissues, as its expression/activity changes with pregnancy and into the postpartum period, and as it modulates hormonal/neurochemical systems and immune processes associated with mood, its deficiency represents a plausible mechanism to partially explain postpartum mood disorders (Davies 2012; Davies 2018).

## Mood abnormalities in steroid sulfatase–deficient individuals

### Human data

Approximately 1 in 750 females from the general population is heterozygous for genetic variants in which the *STS* gene is partially or completely deleted (Trent and Davies 2013; Brcic et al. 2020); as the *STS* gene escapes X-inactivation, these individuals have reduced enzyme activity (Lykkesfeldt et al. 1984). Comprehensive phenotyping of this group has indicated no increased risk of most physical medical conditions, but an increased risk of developmental disorders and associated traits (Cavenagh et al. 2019; Brcic et al. 2020). Outside of the postpartum period, compared to females from the general population, these women also present with elevated levels of mood symptoms including irritability, psychological distress, manic symptoms, and tiredness or low energy, as well as differences with respect to sleeping patterns and weight change (Cavenagh et al. 2019; Brcic et al. 2020). These mood symptoms may be related to a smaller volume of some striatal regions in the brain (Brcic et al. 2020). A study of two female cases with STS deficiency has suggested a possible link to psychotic disorder (paranoid schizophrenia) (Milunsky et al. 1999), although traits related to psychosis were not enriched in larger samples of STS-deficient women (Cavenagh et al. 2019; Brcic et al. 2020).

With respect to the postpartum period, STS-deficient females are more likely than control females to be diagnosed with a mental health condition, notably mild-moderate depression; limited power prevented meaningful analysis of postpartum psychosis rates between groups (Cavenagh et al. 2019). Importantly, the excess of depression-related diagnoses in the STS-deficient group does not seem to be due to increased adverse experiences; moreover, postpartum and non-postpartum depressive traits appear to be dissociable to some extent, suggesting the possibility that STS deficiency in the postpartum period might exacerbate pre-existing non-postpartum psychiatric symptoms (Cavenagh et al. 2019).

### Animal model data

Work in male rodents has shown that deletion of the *Sts* gene, or acute inhibition of the STS enzyme, is associated with numerous behavioural phenotypes including inattention, anxiety-related behaviours, aggression, perseveration, learning and enhanced memory, and behavioural inhibition (Johnson et al. 2000; Nicolas et al. 2001; Davies et al. 2009; Trent et al. 2012b; Babalola et al. 2012; Trent et al. 2013; Davies et al. 2014); these phenotypes may be partially related to serotonergic and cholinergic differences in the hippocampus (Trent et al. 2012a; Trent et al. 2013; Yue et al. 2016). In both rodents and humans, the quality of maternal behaviours is related to maternal executive function (notably offspring-related learning and memory processes, attention to relevant care cues, behavioural flexibility, and impulse regulation) mediated by cholinergic, GABAergic, and serotonergic processes (Davies 2018). Thus, steroid sulfatase appears to influence the mood, behavioural, cognitive, and neurochemical processes associated with effective maternal behaviour.

In female mammals, the idea that STS is associated with postpartum behavioural changes is supported by genetic evidence from a porcine model of relevance to postpartum psychosis where *STS* lies under a candidate quantitative trait locus (Quilter et al. 2007). Additional support comes from a study in which STS was acutely inhibited in new mouse mothers (Humby et al. 2016); these mice showed alterations in various emotional behaviours, i.e. a reduced startle response to an intense acoustic stimulus and altered exploration of an aversive environment. The behavioural abnormalities could be partially alleviated by the administration of the atypical antipsychotic ziprasidone.

Together, the human and animal model data discussed above imply a role for STS in both non-postpartum and postpartum mood symptoms, and suggest that identification of the cellular and molecular pathways disrupted by STS deficiency might indicate novel convergent pathways underlying postpartum mood disorders more generally. Of course, it must be emphasised that risk for these disorders is complex and multifactorial, and that STS-related risk pathways, should they exist, will act in combination with multiple other predisposing and protective factors.

## Molecular mechanisms associated with postpartum symptoms in steroid sulfatase–deficient females

### Perturbed mechanosensory and olfactory function

Whole-brain gene expression analysis in mice in which the STS enzyme was acutely inhibited in the postpartum period identified two genes (*Cyp2g1* and *Stoml3*) which were significantly and robustly upregulated (> 1.5-fold change, *p* < 0.05) (Humby and Davies 2019); these genes both have human orthologues (*CYP2G1P* at 19q13.2 and *STOML3* at 13q13.3). *CYP2G1P* encodes a P450 enzyme important in the olfactory system, although functional alleles in humans are rare (Sheng et al. 2000); functional alleles are expressed throughout the adult brain at low levels (GTEx Portal 2020). *STOML3*, a gene expressed at low levels in the adult human hypothalamus, hippocampus, and basal ganglia (GTEx Portal 2020), encodes a protein important in the gating of Piezo mechanically gated and acid-sensing cation channels and mechanosensory processes through its interactions with membrane cholesterol (Qi et al. 2015; Wetzel et al. 2017); acid-sensing ion channels play a key role in fear and anxiety-related psychiatric disorders (Zha 2013). Although *CYP2G1P* and *STOML3* have not been directly implicated in postpartum psychiatric phenotypes, altered mechanosensory processes may contribute towards depression susceptibility (Howard et al. 2018), and the genomic region around *STOML3* has been associated with autism spectrum disorders, psychotic depression, and schizophrenia risk (Domschke 2013; Li et al. 2016; Roberson-Nay et al. 2018; Bitar et al. 2019). A third gene significantly upregulated in the brain of the STS-inhibited mouse model is *Arhgdig*, encoding the Rho GDP Dissociation Inhibitor Gamma (RhoGDIγ) protein (Humby et al. 2016); the human orthologue of this gene is predominantly expressed in the central nervous system (GTEx Portal 2020) where its protein modulates sensory processes (Ueyama 2019).

Pathway analysis examining less highly differentially expressed genes in the STS-inhibition mouse model (< 1.5-fold, *p* < 0.05) suggested that the expression of genes involved in olfactory signalling were disproportionately affected by the pharmacological manipulation (Humby and Davies 2019); this observation is consistent with a role for steroid sulfates as ligands in the mouse accessory olfactory system (Meeks et al. 2010). In humans, olfactory receptors are expressed in the brain with possible enrichment in certain regions (Flegel et al. 2013) and act as G protein–coupled receptors facilitating the influx of calcium/sodium ions via opening of cyclic nucleotide–gated channels (Nowycky and Thomas 2002; Matthews and Reisert 2003). Whilst heterozygous *STS* deletion has no apparent effect on gross olfactory function (Cavenagh et al. 2019), a more subtle effect cannot be discounted, particularly given that STS inhibition is associated with taste disturbance in female subjects (Stanway et al. 2006).

As the olfactory system and limbic regions extensively connected to it are known to play an important role in mammalian maternal behaviour (Corona and Lévy 2015; Croy et al. 2019), it is plausible that STS deficiency acts to elicit postpartum behavioural symptoms in humans and mice via disturbed olfactory receptor function; potentially, disturbed olfaction could adversely impact upon intermediate phenotypes such as mother-offspring bonding, social judgement, sensory anhedonia, and/or stress modulation. Feasibly, abnormal olfactory processing and the abnormal activity of underlying neural and molecular substrates may play a role in idiopathic postpartum mood disorder risk; consistent with this idea, aberrant olfactory function has been described in several non-postpartum mood conditions (Kamath et al. 2018).

### Disrupted expression and function of Communication Cellular Network factor proteins

The pattern of behavioural effects seen in STS-inhibited postpartum mice signposted a small underlying genetic locus on mouse chromosome 15; brain expression screening of the 17 genes in this interval revealed differential expression (upregulation) of just one, *Ccn3* (encoding Cellular Communication Network Factor 3, previously known as Nephroblastoma-overexpressed or NOV) (Humby et al. 2016). CCN3 can form heterodimers with a second communication cellular network (CCN) protein, CCN2 (previously known as CTGF), and there is some evidence that these two proteins exert antagonistic effects (Davies 2019); *Ccn2* expression is also upregulated in the STS-inhibited postpartum mouse brain (Humby et al. 2016). CCN2 and CCN3 proteins are expressed throughout the cell, but are largely secreted and appear to play an important role in the extracellular matrix (ECM) and multiple associated biological processes including cell proliferation, migration, adhesion, and angiogenesis. CCN2 and CCN3 proteins respond oppositely to mechanical stress in a putative adenylate cyclase–mediated process (Schild and Trueb 2004), and, consistent with this, altered adenylate cyclase type 8 (*Adcy8*) expression is also observed in the STS-inhibited mouse (Humby et al. 2016). CCN proteins are also thought to affect calcium signalling via activation of G protein–coupled receptors, binding to integrins, voltage-gated calcium channels, and/or interaction with intracellular calcium-binding proteins (Lombet et al. 2003). Altered composition or turnover of the ECM has been linked to mood disorders (Lubbers et al. 2014).

Multiple lines of evidence from animal models and clinical studies, summarised previously in Davies (2019), suggest that the CCN2 and CCN3 proteins might be considered as interesting candidate mediators of postpartum mood phenotypes. First, their brain expression is altered in three independent animal models exhibiting postpartum behavioural disturbance, including the STS-inhibition mouse model and the porcine ‘infanticide’ model referred to above (Landers 2019). Second, the CCN3 gene lies directly under a linkage peak identified in a sample of individuals with bipolar affective postpartum psychosis. Third, these genes/proteins are highly expressed in limbic regions of the mammalian brain, show fluctuations in levels through pregnancy and into the postpartum, have been reported to be disrupted in psychotic mood disorders, and mediate relevant behavioural processes including depressive, anxiety-related and neuroticism traits, and sleep. Fourth, of relevance to the idea that precipitous postpartum drops in oestrogen levels stimulate mood changes in some sensitive individuals, oestrogen depletion induces altered CCN3 expression in rat hippocampus, and CCN2 physically associates with the oestrogen receptor to affect its function (Cheng et al. 2011); CCN gene expression is also affected by therapeutic drugs such as antipsychotics and mood stabilisers, and by a range of inflammatory mediators including cytokines, prostaglandins, nitric oxide, histamine, and serotonin. Fifth, altered CCN3 secretion as a consequence of abnormal levels of circulating immunosuppressive regulatory T cells may provide a link between the perturbed inflammatory processes and brain white matter changes linked to postpartum mood (Krause et al. 2014; Duan et al. 2017; Silver et al. 2018; Dazzan et al. 2018). Finally, CCN3 levels are robustly associated with hypertension, and both CCN2 and CCN3 proteins have been strongly implicated in the pathogenesis of pre-eclampsia; hence, their abnormal expression may have pleiotropic effects on behavioural and physiological functions and may partially explain the observed link between postpartum mood disorders and pre-eclampsia.

### Altered balance between sulfated and free steroid hormones

Potentially, an imbalance between sulfated and free steroids in the postpartum period, and the resultant effects on levels of oestrogens and androgens, could explain the STS deficiency effects on behaviour and physiology described above, and might also play a role in idiopathic postpartum mood disorders. Sulfated steroids are produced and secreted in large quantities by the adrenal glands, and dehydroepiandrosterone sulfate (DHEAS) is the most abundant circulating steroid hormone in humans (Neunzig and Bernhardt 2014). Circulating sulfated steroids are rapidly desulfated by STS upon being transported across the blood-brain barrier via organic anion transporter proteins (OATPs) (Grube et al. 2018).

Allopregnanolone (known by the generic drug name brexanolone when produced synthetically), the levels of which are depleted in the postpartum period, and whose restoration in individuals with postpartum depression appears to confer some therapeutic benefit, is metabolised from its sulfate ester by STS, with the former compound exerting effects at GABAergic and NMDA receptors, and on sodium and calcium channels (Johansson and Le Grevès 2005; Viero and Dayanithi 2008; Li et al. 2010; Horishita et al. 2018). Reductions in STS activity will both lower the availability of allopregnanolone and increase the allopregnanolone sulfate to allopregnanolone ratio with consequent effects on neurotransmitter receptor modulation. Levels of a second neuroactive steroid, pregnenolone, have been positively correlated with postpartum anxiety symptoms (Deligiannidis et al. 2016). Like allopregnanolone, pregnenolone also exists in a sulfated form, which exhibits well-characterised activity at the NMDA receptor (Hrcka Krausova et al. 2020); the ratio of pregnenolone sulfate to pregnenolone is, as expected, higher in individuals with STS deficiency (Sánchez-Guijo et al. 2016). Thus, potentially lower absolute pregnenolone levels, or an elevated pregnenolone sulfate to pregnenolone ratio in STS-deficient individuals, may explain changes in anxiety-related behaviours. In addition to effects at the NMDA receptor, pregnenolone sulfate (in contrast to free pregnenolone) potently activates the Transient Receptor Potential Melastatin 3 (TRPM3) calcium ion channel (Wagner et al. 2008), a molecule expressed in adult human brain (GTEx Portal 2020) and recently implicated in neurodevelopmental/neurological conditions (Zhao et al. 2020). Interestingly, genetic variants within the *TRPM3* gene have been associated with abnormal postpartum behaviour in the porcine infanticide model (Bauer 2019) and hippocampal *Trpm3* expression is altered in a mouse model with serotonergic abnormalities and with possible relevance for bipolar disorder (Maddaloni et al. 2018). Functionally, TRPM3 acts as a thermosensitive nociceptor channel implicated in the detection of noxious heat in somatosensory neurons (Thiel et al. 2017); like STOML3, the membrane-linked activity of TRPM3 may be affected by binding to cholesterol (Conrard and Tyteca 2019), and its activity also appears to be sensitive to antidepressant and antipsychotic drugs (Majeed et al. 2008).

## Conclusions and future research avenues

The data from STS-deficient systems discussed above, and summarised in Table 1, provide novel clues as to systems and molecules whose function may be disrupted in postpartum mood disorders. An important question is whether there is any central theme to how these molecules might act; the identification and characterisation of such a nexus might allow targeting by therapeutic drugs.

**Table 1:** Phenotypes of STS-deficient individuals and associated biological mechanisms

Experimental group	Associated phenotypes	Possible underlying biological mechanism(s)
Women heterozygous for genetic deletions including *STS*	Increased risk of developmental and (postpartum) mood disorders	Decreased volume of basal ganglia structures (putamen, globus pallidum, nucleus accumbens)
	Increased self-reported irritability, psychological distress, manic symptoms, and tiredness/low energy	
	Altered sleeping patterns/weight change	
	Rare cases with paranoid schizophrenia	
Male mice with genetic deletions including *Sts*, or with STS enzyme acutely inhibited	Inattention	Increased serotonin (5-HT) levels in the striatum and hippocampus; increased *Htr2c* (serotonin 5-HT 2c receptor) expression in the hippocampus
		Increased hippocampal acetylcholine release
	Increased levels of anxiety-like behaviour, aggression, and perseveration	
		Imbalance between sulfated and free steroids (notably increased dehydroepiandrosterone sulfate (DHEAS) and decreased DHEA)
	Effects on learning and memory	
	Enhanced response inhibition	
Postpartum female mice with STS enzyme acutely inhibited	Decreased acoustic startle response; alleviated by antipsychotic administration	Increased whole-brain expression of *Ccn2*, *Ccn3*, *Adcy8*, *Arhgdig*, *Cyp2g1*, *Stoml3*, and genes involved in olfactory signalling
	Altered exploration of aversive environment	
Postpartum female pigs	Infanticidal behaviour together with anxiety/restlessness in < 10% individuals	Quantitative trait loci overlapping *Sts* and *Htr2c*; increased *Ccn2* and *Htr2c* (long variant) expression in the hypothalamus/brain

**Fig. 1:**
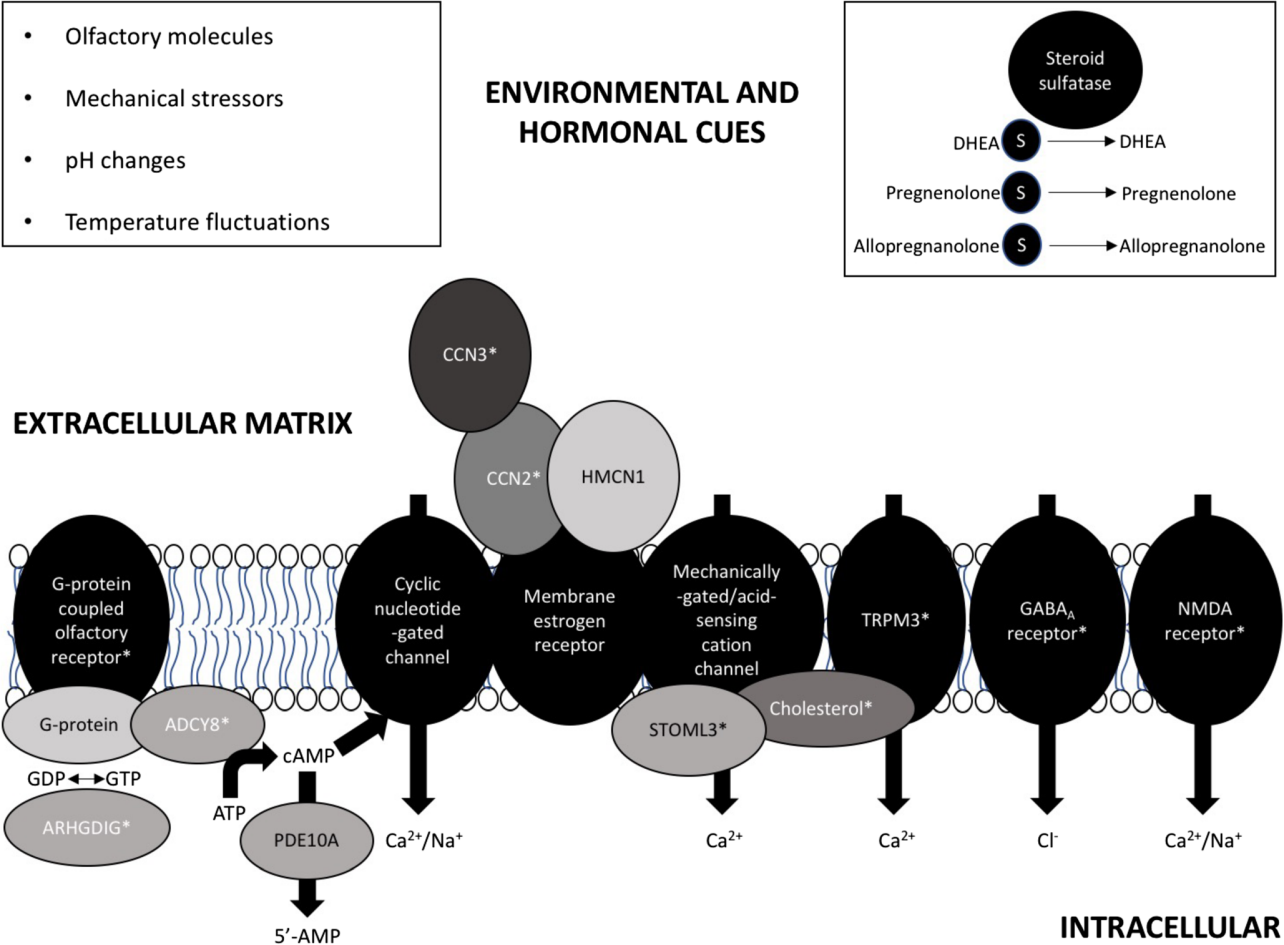
External environmental cues can stimulate neuronal calcium (Ca^2+^) influx via effects on extracellular matrix components (notably CCN proteins and hemicentin (HMCN1)), a variety of transmembrane receptors, and intracellular signalling cascades; the expression/activity of many of these proteins is differentially sensitive to sulfated and free steroids, and/or is affected by steroid sulfatase (STS) deficiency (indicated with an asterisk symbol)

The main theme that emerges is that postpartum mood risk may be a culmination of aberrant steroid hormone modulation of the relationship between extracellular matrix components and membrane receptors involved in perception of the local microenvironment (olfactory, mechanical, pH, and thermal cues) and altered neuronal calcium influx and signalling processes (Fig. 1); consistent with this idea, the gene encoding hemicentin 1 (also known as fibulin-6), an extracellular matrix protein with possible CCN3 binding activity (Vallacchi and Rodolfo 2009) and transmembrane receptor and calcium ion binding activity, has previously been implicated in postpartum mood disorder pathogenesis (Mahon et al. 2009). Brain regions, and their reciprocal connections, of particular interest suggested by the STS work include the olfactory system, the anterior pituitary gland, the lentiform nucleus of the striatum, and the hippocampus; these regions have previously been implicated in postpartum behavioural phenotypes by neuroimaging and histological studies in humans and animal models (Cárdenas et al. 2019; Medina and Workman 2020). Intracellular regulators of calcium influx at cyclic nucleotide–gated channels, e.g. adenylate cyclase and phosphodiesterases (Lane Brown et al. 2006), may also be involved in the pathogenesis of postpartum mood disorders. Abnormal calcium signalling, phosphodiesterase activity, and cyclic AMP/GMP signalling have been implicated in a variety of neurological and psychiatric (mood) conditions (Hebb and Robertson 2007; Harrison et al. 2019), with much focus on phosphodiesterase 10A (PDE10A). PDE10A is highly expressed in the GABAergic neurons of the olfactory tubercle and the striatum (MacMullen et al. 2017), is dysregulated in an animal model of postpartum depression (Yun et al. 2019), and has been genetically associated with bipolar disorder, hypo- and hyperthyroidism, and blood pressure (GWAS Catalog 2020). Although (steroid hormone–dependent) ECM remodelling and calcium signalling have not yet been strongly implicated in postpartum behavioural phenotypes in humans, brain gene expression data in mice have suggested that ‘..signalling, either through the extracellular matrix or transmembrane receptors, is important during pregnancy, parturition, and the postpartum period’ (Ray et al. 2015). Additionally, compared to non-postpartum mice, lactating female mice show gene expression changes in the gonadotropes of the anterior pituitary gland in pathways relating to metal ion transport, cell adhesion, and positive regulation of cGMP metabolic processes (Qiao et al. 2016). Finally, these physiological processes are known to play a prominent role in extra-brain peripartum events including lactation (Cross et al. 2014; Davis 2017), uterine contractions (Loftus et al. 2015), immune system activation (Kimura et al. 2006), and structural changes in the mammary gland, uterus, and ovaries (Thorne et al. 2015).

The ideas discussed above suggest a number of inter-related predictions. Specifically, compared to healthy controls, individuals with idiopathic postpartum mood disorders and relevant animal models may display (a) an elevated ratio of sulfated to free circulating steroids including allopregnanolone, pregnenolone, and cholesterol; (b) genetic association with, and altered expression/activity of, steroid-modulating, extracellular matrix, and signalling molecules (e.g. STS, STOML3, CCN2, CCN3, ADCY8, ARHGDIG, HMCN1, TRPM3, and PDE10A); and (c) evidence for dysregulation of calcium signalling. If any of these predictions are confirmed, the results may inform development of predictive biomarkers and should stimulate further work into identifying specific cellular and molecular risk pathways. Potentially, therapies addressing steroid hormone–dependent perturbations, notably dysfunctional calcium signalling, may be beneficial in treating postpartum mood disorders.

## Data Availability

Not applicable
